# Modified DNA substrate selectivity by GmrSD-family Type IV restriction enzyme BrxU

**DOI:** 10.1098/rstb.2024.0072

**Published:** 2025-09-04

**Authors:** Jennifer J. Readshaw, Yan-Jiun Lee, Peter Weigele, Tim R. Blower

**Affiliations:** ^1^ Department of Biosciences, Durham University, Durham DH1 3LE, UK; ^2^ New England Biolabs, Ipswich, MA 01938, USA

**Keywords:** bacteriophage, DNA modification, restriction enzyme, GmrSD, phage defence, BrxU

## Abstract

Bacteriophages (phages), viral predators of bacteria, generate selection pressure that causes bacteria to evolve defence systems. Type I, II and III restriction enzymes cleave incoming non-modified phage DNAs. Phages have evolved to defend against these restriction systems by modifying their DNA so that they are no longer suitable substrates. Type IV restriction enzymes have evolved to recognize and cleave modified DNA. We have recently characterized and solved the first structure for the Type IV GmrSD-family enzymes, using the BrxU homologue from *Escherichia fergusonii*. Though promiscuous in target modifications, little is known about BrxU substrate preference. We used modified DNAs in *in vitro* assays to characterize the substrate preferences of BrxU and investigate the impact of the GmrSD-inhibitor IPI* on BrxU activity. These data extend our knowledge of phage–host interactions and inform mechanistic studies on the reaction cycle of BrxU and GmrSD homologues.

This article is part of the discussion meeting issue ‘The ecology and evolution of bacterial immune systems’.

## 1. Introduction

Bacteriophages (phages), viral predators of bacteria, generate huge selection pressure by causing an estimated 10^25^ infections of bacterial cells per second [1]. This pressure has caused the evolution of a multitude of bacterial phage defence strategies, which include restriction–modification (RM) systems [2], abortive infection systems [3–5] and CRISPR-*cas* [6]. An increasing diversity of phage defence systems has recently been uncovered owing to the rapid availability of high-quality sequencing data and bioinformatics approaches such as ‘guilt-by-association’. These include BREX [7], CBASS [8], SHIELD [9] and SDIC [10], among others [11–13], many of which utilize conserved protein domains [14]. Defence systems appear to cluster into genetic islands [15], and likely form multi-layered defence networks [16]. Coordinated defence efforts would likely require co-regulation, as was demonstrated by the discovery of the widespread BrxR WYL-domain transcriptional repressors [17–19].

Bioinformatic searches for genes associated with *pglZ*, from the phage growth limitation defence system [20], identified bacteriophage exclusion (BREX) systems [7]. BREX defences are some of the most prevalent phage defence systems, encoded within approximately 10% of bacterial and archaeal genomes [7,21]. Together with *gmrS*/*gmrD*, which encode a Type IV restriction enzyme [22], BREX genes form one of the most common defence island pairings [23,24]. We have recently demonstrated that BREX and a GmrSD homologue, BrxU, encoded on a defence island of a multidrug-resistant plasmid of *Escherichia fergusonii*, provide complementary phage defence [15]. Of the six BREX sub-types, Type I BREX contains six conserved genes: *brxA*, *brxB*, *brxC*, *pglX*, *pglZ* and *brxL* [7]. BrxA is a DNA-binding protein [25]. BrxL is a DNA-stimulated AAA+ ATPase [26]. The PglX methyltransferase targets non-palindromic 6 bp sequences (BREX motifs) on the N6 adenine at the fifth position of the sequence, distinguishing host from foreign DNA [7,15,27–30]. Though reminiscent of RM, the mechanism of how BREX acts upon DNA with non-modified BREX motifs is not known. In contrast, BrxU is a Type IV restriction enzyme that recognizes and cleaves modified DNA [15,22].

Early studies of the T-even phages, T2, T4 and T6, identified the inclusion of modified bases such as 5-hydroxymethylcytosine (5hmdC) in bacteriophage genomes [31]. The range of modified [32] and hypermodified [33] bases has since expanded. Diverse modifications have been characterized in dG, dA, dC and dT bases [34,35], and some phages can fully replace dT with dU [36]. DNA modifications can protect injected phage DNA from being targeted by defence systems, including restriction enzymes [37], BREX [30] and CRISPR-*cas* [38]. However, defence systems have evolved that specifically recognize and degrade modified DNAs [39].

Type IV restriction enzymes, first characterized through the *mcrA* and *mcrBC* systems (*m*odified *c*ytosine *r*estriction), are a disparate set of enzymes gathered together by functionality. They differ in the modifications recognized, their conserved amino acid sequences, their structure and how they cut the modified DNA [39]. The GmrSD family of Type IV enzymes was identified on a prophage of *Escherichia coli* CT596 as two separate genes encoding GmrS and GmrD [40], though a single-gene version encoding a fused GmrS-GmrD polypeptide was subsequently shown to be more prevalent [22]. Studies of one such single polypeptide Eco94GmrSD homologue demonstrated that substrate degradation could use a broad range of nucleotide cofactors [41]. Subsequent studies of another fused GmrS-GmrD homologue, BrxU, produced the first structures of this family and demonstrated that nucleotide binding causes monomerization of GmrSD dimers [15]. While GmrSD homologues provide effective phage defence, phages such as T4 encode an injectable protein inhibitor, IPI*, which blocks GmrSD activity [42,43].

Previously, we demonstrated that BrxU recognizes multiple cytosine modifications, but GmrSD-family substrate preferences for specific DNA modifications have not been fully characterized [15]. We therefore performed *in vitro* assays to characterize the substrate preferences of BrxU and investigate the impact of IPI* on BrxU activity.

## 2. Results

### (a) BrxU cleaves DNA with a broad range of cytosine modifications

**Figure 1.**
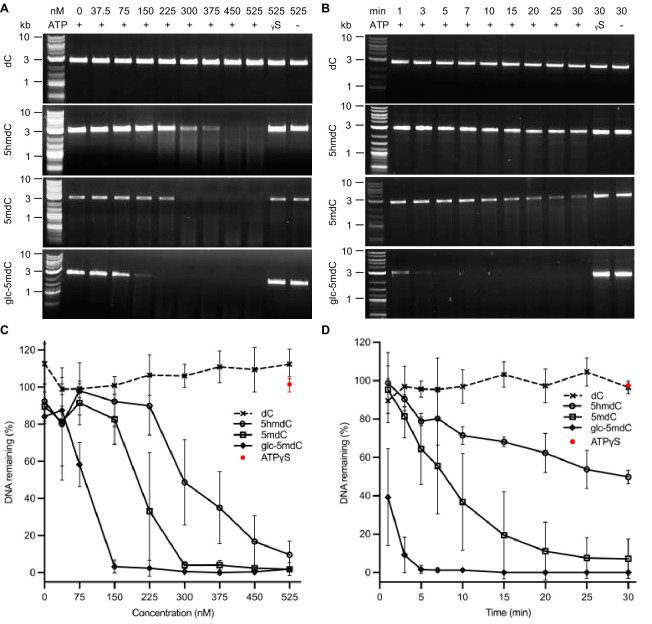
BrxU demonstrates preference between differing cytosine modifications. (A) Titrations of His_6_-BrxU were incubated with 100 ng of pUC19 incorporated with dC, 5hmdC, 5mdC or glc-5mdC for 10 min. The reactions were performed in triplicate, and representative gels are shown. The presence of ATP in the reaction is denoted with a + or − sign, and ATPγS is denoted by γS. (B) Timecourse of His_6_-BrxU (300 nM) mixed with 100 ng of pUC19 incorporated with dC, 5hmC, 5mC or glc-5hmC and incubated at 37 °C for from 1 to 30 min. The reactions were performed in triplicate, and representative gels are shown. The presence of ATP in the reaction is denoted with a + or − sign, and ATPγS is denoted by γS. (C) The average percentage of DNA remaining in His_6_-BrxU titration reactions with ATP compared with reactions without ATP, calculated from triplicate gels. Error bars represent standard deviation. (D) The average percentage DNA remaining in His_6_-BrxU timecourse reactions with ATP compared with reactions without ATP, calculated from triplicate gels. Error bars represent standard deviation.

Previously, we demonstrated that BrxU can cleave DNA containing 5-methyl cytosine (5mdC), 5hmdC and glucosyl-5-oxymethyl cytosine (glc-5mdC) modifications [15]. However, we had no data on substrate preference. We therefore generated modified DNA substrates using modified bases in a PCR amplifying pUC19. These substrates are identical in size, sequence and number of modifications (equal to the number of cytosines), but differ in the type of modification. The resulting substrates were used to examine BrxU activity against cytosine modifications through titration and during a timecourse (figure 1). Based on titrations, glc-5mdC is the preferred substrate, followed by 5mdC and then 5hmdC (figure 1A,C). Digestion by BrxU was blocked in the absence of ATP or the use of non-hydrolysable analogue ATP-γ-S (figure 1). Timecourse assays showed a distinct preference for glc-5mdC, with the bulk of the substrate degraded within the first minute of incubation, followed much more slowly by 5mdC and then 5hmdC (figure 1B,D).

### (b) Phage counter-defence IPI* can inhibit BrxU

**Figure 2.**
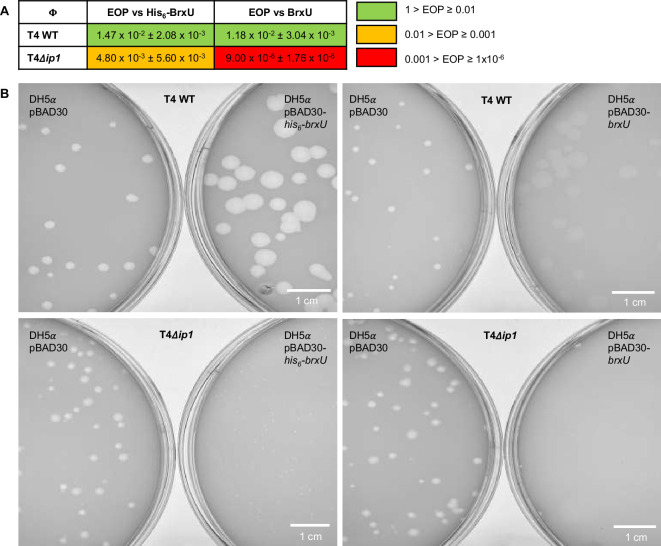
BrxU phage defence can be inhibited by T4 IPI* counter-defence. Phages T4 WT and T4Δ*ip1* were plated on *E. coli* DH5α pBAD30-*his*_*6*_*-brxU* or DH5α pBAD30-*brxU* and compared against DH5α pBAD30. (A) EOP values for each phage are shown; values are mean EOPs from triplicate data with standard deviation. (B) Example images of plaques are provided.

Eco94GmrSD was shown to be inhibited by IPI*, a protein stored in the capsid of T4 and injected along with the phage genomic DNA [40–43]. Full-length IPI is encoded by *ip1*, and the product is post-translationally cleaved by the head assembly protease Gp21 to make the 76 amino acid active form, IPI* [43]. We tested the ability of BrxU to block plaquing by T4 wild-type (WT) and a T4Δ*ip1* mutant (figure 2). By efficiency of plating (EOP), His_6_-BrxU had only a modest quantitative impact on T4 WT of approximately 100-fold reduction (figure 2A). Curiously, the size of the T4 plaques was larger than on the pBAD30 control strain (figure 2B). When tested against T4Δ*ip1,* there was a further small drop in EOP against His_6_-BrxU (figure 2A), and plaque size substantially decreased to pinpricks (figure 2B). This indicated that IPI* could provide some inhibition of His_6_-BrxU activity in cells. We then tested how untagged BrxU would act against the two phages. With T4 WT, BrxU again provided approximately 100-fold reduction in EOP and an increase in plaque size, but the plaques were cloudy (figure 2). With T4Δ*ip1*, BrxU had a greater impact than His_6_-BrxU, causing a 6-log reduction in EOP and again changing plaque size to pinpricks (figure 2). These data indicate that IPI* is better able to inhibit BrxU than His_6_-BrxU.

**Figure 3.**
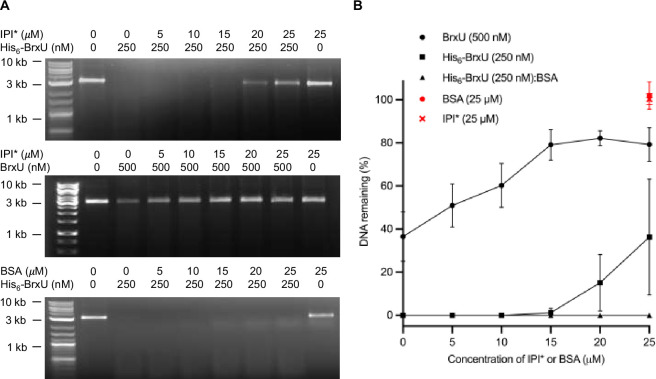
*In vitro* inhibition of BrxU by IPI*. (A) His_6_-BrxU and BrxU samples incubated with IPI* or BSA for 30 min were further incubated with 100 ng glc-5mdC pUC19 and 1 mM ATP for 10 min. The products were resolved on 1% agarose gels. Reactions were performed in triplicate, and representative gels are shown. (B) The mean average percentage DNA remaining in reactions of His_6_-BrxU and BrxU with IPI* or BSA, calculated from triplicate gels. Error bars represent standard deviation.

Our observed EOP phenotype for His_6_-BrxU was not as impressive as the inhibition of Eco94GmrSD in EOP assays by IPI*, where the deletion of *ip1* from T4 caused a 2−4 log reduction in EOP [41]. However, using untagged BrxU in cells appeared to show the same effect as for Eco94GmrSD. We therefore aimed to show *in vitro* inhibition of both His_6_-BrxU and BrxU by IPI*. We were able to express and purify IPI*, and then titrated IPI* against His_6_-BrxU and BrxU during digestion of glc-5mdC pUC19 DNA (figure 3). We observed inhibition of His6_6_-BrxU in the presence of around 80−100 times molar excess of IPI*, whereas a 30-fold excess of IPI* was sufficient to provide robust inhibition of BrxU (figure 3A,B). To check whether an abundance of non-specific protein would have a similar impact, we also performed the titration using bovine serum albumin (BSA) as a control and saw no alteration of His_6_-BrxU activity (figure 3A,B). These data show that IPI* inhibits BrxU activity during phage infections, and that BrxU is specifically inhibited by IPI* within *in vitro* DNA cleavage assays.

## 3. Discussion

Phages have evolved to modify their genomes, and this provides protection from some forms of restriction. Type IV restriction enzymes have evolved to cleave modified DNA. Phages have, in return, evolved inhibitors of Type IV enzymes. In this study, the GmrSD-family of Type IV enzymes, represented by BrxU, was investigated for substrate preference and inhibition by phage protein IPI*.

GmrSD homologues have thus far been shown to target cytosine modifications [15,41]. Fully glycosylated DNA, glc-5mdC, was the clear preferred substrate by titration and timecourse (figure 1). The next preferred was 5mdC, while 5hmdC was the least preferred, implying that in some way the hydroxyl reduces BrxU activity (figure 1). The other GmrSD homologue to be characterized biochemically, Eco94GmrSD, demonstrated cleavage of 5hmdC, but was not tested against other substrates [41]. Though the structure of BrxU has been solved crystallographically [15], this was done in the absence of DNA, and so it is unclear how a DNA modification would be recognized, but clearly there must be volume to accommodate larger modifications such as glycosylation. Further structural biology studies should be performed using modified substrates and a nuclease-deficient mutant in order to better understand substrate recognition—be it through flipping of the base, binding in a specific pocket, or another as yet undescribed mechanism. An initial starting point could be to co-crystallize or soak crystals with glucose, as a means to position likely modification binding pockets, should they exist.

Eco94GmrSD provided *E. coli* with only weak protection against T4, about 15- to 20-fold [41]. This was attributed to the release of IPI* during T4 infection. Subsequent infection assays using mutant T4Δ*ip1* saw plating reduced by 10^−6^ to 10^−7^, implying the Eco94GmrSD could now provide robust protection [41]. Though His_6_-BrxU and BrxU provided similarly poor protection from T4 WT as had Eco94GmrSD (figure 2A), when using T4Δ*ip1,* we did see further reductions in EOP, indicating that partial His_6_-BrxU and BrxU protection was restored (figure 2A). The quantitative changes were supported by clear qualitative changes, in that plaques of T4Δ*ip1* were much smaller in the presence of His_6_-BrxU and BrxU (figure 2B). Notably, His_6_-BrxU can protect against some phages in the order 10^−9^ [15]. As T4 has glc-5mdC modified DNA, it was therefore unexpected that the His_6_-BrxU phenotype against T4Δ*ip1* was so minimal. When we tested untagged BrxU, we saw greater activity against T4Δ*ip1*, indicating the tag might have some impact on BrxU activity specifically against T4, and this should be investigated further.

Owing to the weak *in vivo* phenotype, we chose to perform an *in vitro* inhibition assay using purified IPI* (figure 3). Compared with a BSA control, we observed IPI* causing direct inhibition of both His_6_-BrxU and BrxU activity, proving IPI* is an inhibitor of BrxU. We also noted that IPI* inhibition was more effective against untagged BrxU. The differences between the impact of IPI* against Eco94GmrSD and our phenotype against BrxU are not unprecedented, as IPI* has been previously shown to have distinct specificity for GmrSD homologues [42]. When tested, AlphaFold predictions of BrxU and Eco94GmrSD dimers correlated poorly with the solved crystallographic structure of BrxU, and predictions of IPI* : BrxU complexes were similarly poor. It will be necessary to first characterize IPI* : BrxU complexes, if required altering the combinations of homologues used, before again performing structural studies to understand IPI* inhibition. These data will provide further information towards understanding the complex GmrSD cycle of substrate recognition and cleavage. In turn, this information provides a deeper understanding of phage–host interactions and the use of Type IV restriction enzymes in selective recognition and cleavage of modified DNA.

## 4. Material and methods

### (a) Bacterial strains and culture conditions

*E. coli* strain DH5α was sourced from Invitrogen. *E. coli* Rosetta 2(DE3)pLysS was sourced from ThermoFisher Scientific. All strains were grown at 37 °C, either on agar plates or shaking at 150 r.p.m. for liquid cultures. For liquid cultures, 2× YT was used as the standard growth medium, and Luria Broth (LB) was supplemented with 0.35% w/v or 1.5% w/v agar for semi-solid and solid agar plates, respectively. Growth was monitored using a spectrophotometer (WPA Biowave C08000) measuring optical density at 600 nm (OD_600_). When necessary, growth media were supplemented with ampicillin (Ap, 100 µg ml^−1^), spectinomycin (Sp, 100 µg ml^−1^), chloramphenicol (25 μg ml^−1^), isopropyl-β-d-thiogalactopyranoside (IPTG, 1 mM), d-glucose (0.2% (w/v)) or l-arabinose (l-ara, 0.1% (w/v)).

### (b) Use of coliphages

Phages T4 and T4Δ*ip1* were sourced from New England Biolabs [41], and lysates were produced using *E. coli* DH5α. To make lysates, 10 μl of phage dilution was mixed with 200 μl of *E. coli* DH5α overnight culture and mixed with 4 ml of sterile semi-solid ‘top’ LB agar (0.35% agar) in a sterile plastic bijou. Samples were poured onto solid LB agar plates (1.5% agar) and incubated overnight at 37 °C. Plates showing a confluent lawn of plaques were chosen for lysate preparations, and the semi-solid agar layer was scraped off into 3 ml of phage buffer. To each sample, 500 μl of chloroform was added, and samples were vigorously vortexed and incubated for 30 min at 4°C. Samples were centrifuged at 4000*g* for 20 min at 4 °C, and the supernatant was carefully transferred to a sterile glass bijou. Then, 100 μl of chloroform was added, and lysates were kept at 4°C for long-term storage.

### (c) Protein expression and purification

Production of His_6_-BrxU and untagged BrxU was performed as described using plasmid pTRB519 and pTRB446, respectively [15]. For production of IPI*, the mature form open reading frame of *ip1* (amino acids 20−95 inclusive) was synthesized and cloned into pBAD30 [44] encoding a cleavable His_6_-SUMO tag from pSAT1-LIC [45], by Genscript. For expression of IPI*, *E. coli* DH5α was transformed with the pBAD30-His_6_-SUMO-IPI* expression construct. A single colony was used to inoculate 100 ml 2× YT containing ampicillin (Ap) and grown overnight at 37^o^C. Overnight cultures were used to inoculate six 2 l baffled flasks containing 1 l of 2× YT supplemented with Ap. Cultures were grown at 37^o^C, shaking at 150 r.p.m., until OD_600_ of approximately 0.6, at which point, expression was induced by the addition of l-ara to a final concentration of 0.1% (w/v), and cultures were grown overnight at 18 ^o^C. Cultures were pelleted at 5000*g* for 30 min at 4^o^C. Pellets were resuspended in 25 ml of ice-cold A500 (20 mM Tris-HCl pH 7.9, 500 mM NaCl, 10 mM imidazole and 10% glycerol v/v) and lysed via sonication, and the lysate was centrifuged at 45 000*g* for 1 h at 4^o^C. The clarified cell lysates were passed over a 5 ml HisTrap HP column (Cytiva), washed with 50 ml A100, and eluted directly onto a HiTrap Q HP column (Cytiva) with B100 (20 mM Tris-HCl pH 7.9, 100 mM NaCl, 250 mM imidazole and 10% glycerol v/v). The column was transferred to an Äkta Pure (Cytiva) fast-protein liquid chromatography (FPLC) system for anion exchange chromatography (AEC) and eluted with a gradient from 100% A100 to 60% C1000 (20 mM Tris-HCl pH 7.9, 1 M NaCl, 10 mM imidazole and 10% glycerol v/v). Fractions containing the target protein were pooled and incubated with 200 μl of 2 mg ml^−1^ hSENP2 SUMO protease overnight at 4^o^C to cleave the His_6_-SUMO tag [45]. Untagged IPI* was passed over a second HisTrap HP column and the flowthrough was collected, concentrated using a 3 kDa molecular weight cut-off concentrator (Thermo Scientific), and resolved by size-exclusion chromatography (SEC) through a Sephacryl S-200 HR gel filtration column using SEC buffer (50 mM Tris-HCl pH 7.9, 500 mM KCl, 10% (v/v) glycerol). Fractions were analysed by SDS-PAGE, and peak fractions were pooled. IPI* was stored in 30% (v/v) glycerol at −80^o^C.

### (d) BrxU assays

Synthetically modified substrates were produced by PCR using pUC19 as a template. Amplification was carried out replacing deoxycytidine triphosphate (dCTP) with either 5hmdC or 5mdC (Jena Biosciences). The 2.7 kb amplicon was purified using a Monarch PCR Cleanup Kit (NEB). Amplicons containing 5hmdC were treated with T4 phage bβ-glucosyltransferase (NEB) using UDP-glucose as a donor to produce glc-5mdC. His_6_-BrxU and BrxU titration assays with IPI* were set up with 1 mM ATP, 1× BMG buffer (20 mM Tris-HCl pH 7.5, 50 mM CH_3_COOK, 10 mM MgSO_4_) and 100 ng of 2.7 kb pUC19 substrate or phage genomic DNA (gDNA). Reactions were carried out at 37^o^C for 15 min. Timepoint assays were set up under the same conditions, but reactions were carried out for up to 30 min. Negative controls used ATPγS or nuclease-free water in place of ATP, or BSA (Sigma) in place of IPI*. All reactions were terminated by incubating at 75^o^C for 10 min.

Agarose gels composed of 1.0 or 1.8% agarose containing 0.004% (v/v) ethidium bromide were resolved at 80 V for 45 min in 1× TAE buffer. All gels were run in triplicate and visualized with Bio-Rad Image Lab software. The remaining substrate was quantified by comparison with the no ATP control using FIJI ImageJ. Densitometry was performed with background subtracted. The area to be measured in each lane was selected based on the location of the intact control DNA, and histograms were plotted in which the area of each histogram represented the DNA band intensity in the respective lane. The average intensity of the intact DNA in the control lane was normalized to 100%, against which the average DNA intensity in the test lanes was compared.

### (e) Efficiency of plating assays

*E. coli* DH5α were transformed with pBAD30 (vector control) [44], pTRB519 (pBAD30-*his_6_-brxU*) [15] or pTRB768 (pBAD30-*brxU*), generated by the removal of the His tag from pTRB519 (Genscript). Serial dilutions of phages T4 and T4Δ*ip1* were produced in phage buffer (10 mM Tris-HCl pH 7.4, 10 mM MgSO_4_, 0.01% (v/v) gelatin). To 3 ml of 0.35% LB agar, 200 µl of overnight culture and 10 μl of a phage dilution were added. Plates were incubated overnight at 37°C before plaque-forming units (pfu) were counted on each plate. The EOP was calculated by dividing the pfu of the test strain by the pfu of the control strain. Data shown are the mean and the standard deviation of at least three biological and technical replicates.

## Data Availability

All data needed to evaluate the conclusions in the paper are present in the paper.
